# Complete mitochondrial genome and phylogenetic position of *Chaenodraco wilsoni*

**DOI:** 10.1080/23802359.2017.1372712

**Published:** 2017-08-30

**Authors:** Yao Dong, Chunyan Ma, Fengying Zhang, Liyan Qu, Chunlei Feng, Wei Song, Luming Wang, Lingbo Ma

**Affiliations:** a Key Laboratory of East China Sea and Oceanic Fishery Resources Exploitation, Ministry of Agriculture, East China Sea Fisheries Research Institute, Chinese Academy of Fishery Sciences, Shanghai, China;; b College of Fisheries and Life Sciences, Shanghai Ocean University, Shanghai, China

**Keywords:** *Chaenodraco wilsoni*, mitochondrial genome, phylogenetic analysis

## Abstract

*Chaenodraco wilsoni*, a species of Channichthyidae, inhabits in southern ocean. The total length of complete mitochondrial genome of *C. wilsoni* is 17,432 bp, which encoded 37 genes. Similar to most Antarctic fishes, the ND6/tRNA (glu) translocation and an additional non-coding region linked with ND6 have also occurred in *C. wilsoni*. The ML tree supports that *C. wilsoni* has closer relationship with *Chionodraco* species. Our research will provide more molecular biology information about *C. wilsoni* and deepen the understanding of Antarctic fishes.

*Chaenodraco wilsoni*, an important spiny icefish, is distributed in southern ocean. It is a kind of benthic fish. The adult fish mainly inhabits 200–800 m-depth water, however, the post-larvae and juveniles inhabit 100 m-depth water (Iwami and Kock 1990; Mesa et al. 2009). In the eastern Ross Sea, *C. wilsoni* composed 8% benthic biomass (Donnelly et al. 2004). In order to exploit Antarctic resources scientifically, the comprehensive understanding of Antarctic fish is necessary.

The *C. wilsoni* was collected from South Shetland Islands (60°59′S, 63°57′W) on 10 February 2014. Fin tissues were sampled and preserved in −20 °C for further DNA extraction. The DNA is stored in East China Sea Fisheries Research Institute, China. Referring to the sequence of related species *Chionodraco hamatus* (KT921282.1), primers were designed for amplifying and sequencing the complete mitochondrial genome of *C. wilsoni*.

The complete mitochondrial genome of *C. wilsoni* was 17,432 bp (GenBank No. MF536715), which encoded 13 protein-coding, two rRNA and 22 tRNA genes. Moreover, a putative control region was observed. Similar with most fishes (Lee et al. 2015; Soon et al. 2016), AT bias (A + T 52.66%) also appeared in *C. wilsoni*. The ND6 was started by ATG but ended by AGG, which is an alternative termination codon in vertebrate mitochondrion (Lee et al. 2015). The remaining 12 protein-coding gene was started by ATG or GTG and ended by TAA, TAG, TA-, or T-, respectively. In the mitochondrial genome of *C. wilsoni*, ND6/tRNA(glu) translocation and an additional non-coding region, closely linked with ND6, were consistent with most Antarctic fish, such as *Chionodraco hamatus, Chaenocephalus aceratus* (Lee et al. 2015), and *Notothenia coriiceps* (Soon et al. 2016).

To identify the phylogenetic status of *C. wilsoni* in Perciformes, the maximum likelihood (ML) tree was built using the online web RAXML (http://www.ch.embnet.org/raxml-bb/) based on 12 protein-coding genes (expect ND6) (Stamatakis 2014), with *Cyprinus carpio* as an outgroup (Figure 1). The 20 species were mainly clustered into three big branches. *C. wilsoni* forms a sister group with *Chionodraco hamatus* and *Chionodraco myersi*. Then, this group combined with the remaining Channichthyidae species and Bathydraconidae, formed an ultimately monophyletic group, which was consistent with Eastman who had studied the phylogenetic relationship of Notothenioid fishes based on body morphology (Eastman et al. 2014).

**Figure 1.**
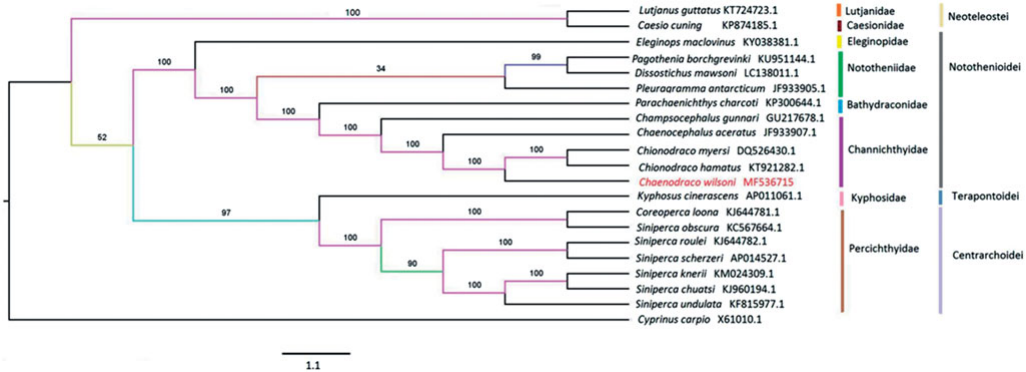
Maximum likelihood (ML) tree based on the mitochondrial 12 protein-coding genes (except ND6) of *C. wilsoni* and other 19 related species, *Cyprinus carpio* (X61010.1) used as an outgroup species.

Our study firstly obtained the complete mitochondrial genome of *C. wilsoni* and analysed its phylogenetic position in Perciformes. This will provide more molecular biology information about *C. wilsoni* and deepen the understanding of Antarctic fishes.
